# Comparison of fungal and bacterial keratitis between tropical and subtropical Taiwan: a prospective cohort study

**DOI:** 10.1186/s12941-020-00353-3

**Published:** 2020-03-30

**Authors:** Chih-An Chen, Shiuh-Liang Hsu, Ching-Hsi Hsiao, David Hui-Kang Ma, Chi-Chin Sun, Hun-Ju Yu, Po-Chiung Fang, Ming-Tse Kuo

**Affiliations:** 1 Department of Ophthalmology, Kaohsiung Chang Gung Memorial Hospital and Chang Gung University College of Medicine, Kaohsiung City, Taiwan; 2 Department of Ophthalmology, Kaohsiung Medical University Hospital and Kaohsiung Medical University, Kaohsiung City, Taiwan; 3 Department of Ophthalmology, Linkou Chang Gung Memorial Hospital and Chang Gung University College of Medicine, Taoyuan City, Taiwan; 4 Department of Ophthalmology, Chang Gung Memorial Hospital, Keelung and Department of Chinese Medicine, Chang Gung University, Taoyuan, Taiwan

**Keywords:** Corneal ulcer, Fungal keratitis, Bacterial keratitis, Climates

## Abstract

**Background:**

Fungal keratitis (FK) has been shown to be a climate-sensitive disease. The differentiation between FK from bacterial keratitis (BK) was difficult. The purpose of this study was to compare the bacteriology and mycology between tropical and subtropical Taiwan and to investigate the independent risk factors for identification of fungi from bacteria.

**Methods:**

Two hundred ninety-seven patients with clinical suspected microbial keratitis were prospectively enrolled. A fungal to bacteria rate (FBR), the number of fungi divided by bacteria identified, was determined to estimate the prevalence of fungi and bacteria. Clinical presentation, profiles of microorganisms, and predisposing risk factors were determined. Univariate and multivariate logistic regression analysis were used to investigate the independent risk factors.

**Results:**

A total of 82 fungi and 143 bacteria were laboratory confirmed. The identification rate of fungus was higher in tropical Taiwan (*p *= 0.010). Among the fungi and bacteria confirmed, the FBR was 0.29 (22.4% vs. 77.6%) in subtropical Taiwan, and 0.70 (41.3% vs. 58.7%) in tropical Taiwan. Samples obtained in tropical area (*p *= 0.019), ocular trauma (*p *= 0.019), and plant exposure (*p *= 0.003) were independent risk factors for identification of fungus from bacteria. The predominant fungus isolated from corneal scraping were *Fusarium solani* (25%) and *Trichosporon faecale* (25%) in subtropical Taiwan; in tropical Taiwan was *Fusarium* spp. (50%).

**Conclusions:**

The identification rate of fungus was higher in tropical Taiwan than subtropical Taiwan. Awareness of the local epidemiology is crucial for early diagnosis of fungal keratitis in tropical area.

## Background

Microbial keratitis (MK) is a major cause of monocular blindness, especially in developing country [1]. Among MK, fungal keratitis (FK) is a climate-sensitive, severe sight-threatening infectious disease, and its prognosis is worse than bacterial keratitis (BK). In developed countries, the incidence of FK is also in a rising trend [2]. However, the suppuration of cornea may mask the clinical characteristics of FK, making the differentiation between early stage FK from BK more difficult [3]. Delayed diagnosis is common in FK because a positive fungal culture requires a long time. Therefore, it is critical to characterize the epidemiology, the demographic data, and risk factors for FK to aid the ophthalmologists to make early diagnosis and prompt treatment.

The proportion of FK increases as the tropical latitudes increases [4]. Regarding to agricultural work associated plant exposure [1, 5], different climate types [5, 6], low income status [7], extent of urbanization [8], and public health conditions, about 37.6% to 60.6% of cases of corneal ulcer had a mycolic etiology [1, 4, 9, 10], especially in tropical and subtropical area. According to the variation of climate types and public health status, the fungal to bacteria rate (FBR) in subtropical [8] and tropical area [8, 11] range widely from 0.2 to 0.5 and 0.5 to 2.1, respectively. However, there is a paucity of studies that directly compare the difference between subtropical and tropical area in the same country. To date, most large-scale epidemiologic studies on MK in Taiwan were performed in subtropical area [12–14]. These local information cannot be generalized to different geographical locations and climate zones.

Tropic of Cancer divides Taiwan into tropical and subtropical zone. This is also the dividing line for climate as equatorial savannah with dry winter (Aw) and warm temperate climate with fully humid (Cfa) types, defined by the Koeppen–Geiger climate classification [15]. Subtropical and tropical Taiwan had similar baseline characteristics, including racial, demographic profile, occupational and socio-economic status, income class, health care system, and extent of urbanization, with climate classification being the only variability. Therefore, we designed a prospective multi-center study to compare the cases of presumed MK in tropical and subtropical Taiwan over a 2-year period. The mycology, bacteriology and predisposing factors to fungal identification were determined.

## Methods

### Participants

This prospective multi-center cohort study was conducted in Taiwan to investigate the mycology and bacteriology of patients with MK in different climate zones. The participating research medical centers were as follows: Keelung Chang Gung Memorial Hospital (Keelung, subtropical Taiwan), Linkou Chang Gung Memorial Hospital) (Taoyuan, subtropical Taiwan), Kaohsiung Chang Gung Memorial Hospital (Kaohsiung, tropical Taiwan), and Kaohsiung Medical University Hospital (Kaohsiung, tropical Taiwan). Patients with clinically suspected MK were enrolled from the emergency department and outpatient clinic in the listed hospitals from January 1, 2015 to December 31, 2016, and were followed up for at least 6 months. The inclusion criteria were presence of corneal epithelial defect with infiltration. On first visit, patients were required to undergo the pre-registration evaluation, including the demographic data, ocular and systemic risk factors, slit lamp examination, record of clinical presentation of MK, and collection of corneal scrapes. All eligibility data with the written consent form and specimens for molecular tests were sent to Kaohsiung Chang Gung Memorial Hospital data center (M.T.K.), and was reconfirmed that met the inclusion criteria. Finally, patients with presumed MK were registered as confirmed eligible. During follow-up period, the data of microbial culture results were collected.

This study adhered to the Declaration of Helsinki and the protocol was approved by the Institutional Research Ethics Board at Chang Gung Memorial Hospital (IRB number: 103-0640B). During data collection, the authors had access to information that could identify individual participants. Written informed consent was obtained from each participant for inclusion in the study. A total of 297 corneal scraping samples were prospectively collected.

### Microbiological investigation

Corneal scrapes were collected by ophthalmologists with a no. 15 sterile microsurgical blade. For these patients, a comprehensive microbial survey was arranged to examine all specimens from patients with presumptive MK, including conventional standard tests (microscopy with Gram stain, potassium hydroxide (KOH) wet mount, and culture) and molecular tests [Polymerase chain reaction (PCR) and/or dot hybridization assay (DHA)]. For a corneal ulcer with diameter more than 2 mm, the specimens were equally divided for smear, culture and PCR; for a corneal ulcer with diameter less than 2 mm, the specimens were divided for culture and PCR [16]. The laboratory confirmation of FK or BK was defined as positive smear, culture, or PCR/DHA results.

For fungal identification, after being smeared onto 10% KOH wet mount and acid-fast staining, the corneal scrape was inoculated onto Sabouraud’s agar, and then incubated at 30 °C for 4 weeks. For bacterial identification, the corneal scrape was smeared onto Gram staining. The standard culture system involved blood agar, eosin methylene blue agar, and chocolate agar, with incubation at 35 °C for 72 h in a humidified *atmosphere* containing 5% CO_2_. After incubation, the obtained microbial culture was identified with standard biochemical tests.

Molecular tests included amplification of target genes with PCR and/or following DHA. If the PCR result was negative, DHA was additionally performed. Standard validated fungal [16] and bacterial [17] primer sets were used for PCR. For dot assay, 10 μL of PCR product were subjected to hybridization to a fungus- [16] or bacteria- [17] specific oligonucleotide probe on a nylon membrane.

### Statistical analysis

The demographic characteristics and etiology of the participants, the proportion of fungi and bacteria identified in tropical and subtropical Taiwan were compared with Pearson’s chi-square test for categorical variable and Student *t* test for continuous variable in normal distribution. The isolated pathogens, especially for those bacteria and fungi confirmed to the genus level were listed. Due to all the data were collected on first visit, there was no loss to follow-up bias in this study.

To determine the independent risk factors for the identification of fungus in fungal or bacterial microorganism identified, multivariate logistic regression was applied to calculate the odds ratio (OR) and 95% confidence intervals (*CI*). Only patients with positive fungal or bacterial identification were analyzed. Co-linearity between multiple variables was diagnosed using variance inflation factor (VIF). Poisson regression was used to assess the effect of average monthly temperature and precipitation on the number of fungal or bacterial identification. Statistical significance was considered as *p* value < 0.05. The statistical analyses were conducted using SPSS software, version 20 (IBM, Armonk, NY).

## Results

**Fig. 1:**
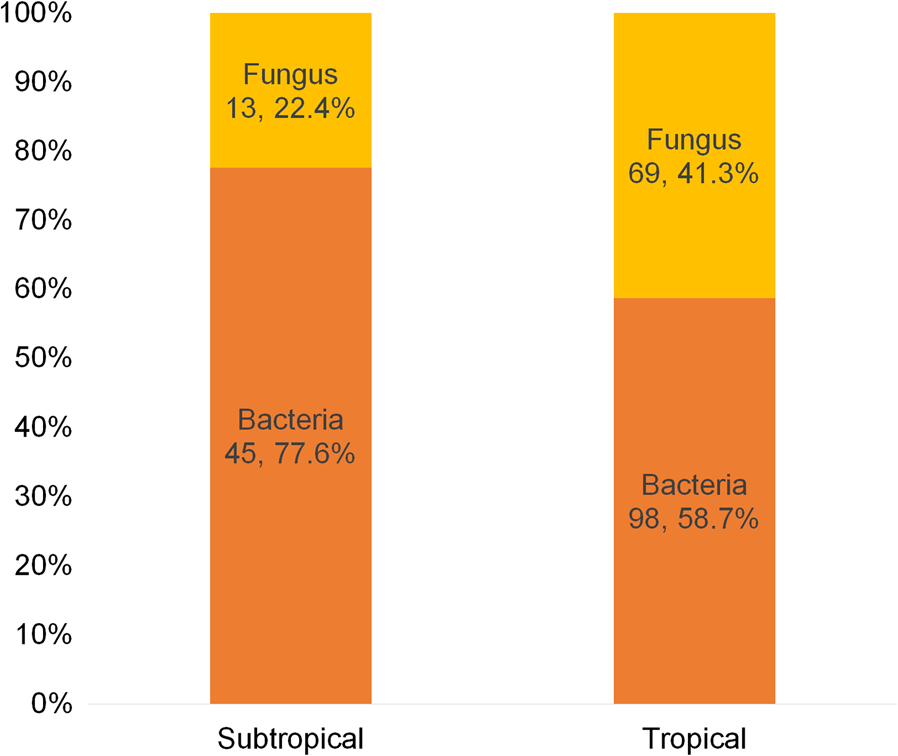
Number of Bacteria or Fungi Identified in Subtropical and Tropical Taiwan from Corneal Scarping. Among bacteria and fungi identified, there were significant differences in proportion of number of fungi (22.4% vs. 41.3%, p = 0.010) and bacteria (77.6% vs. 58.7%, p = 0.010) between subtropical and tropical Taiwan. Significance was determined by the Pearson’s Chi square test

During the 2-year period, 297 patients were confirmed eligible and were included in this study, of which 64 and 233 were from subtropical and tropical Taiwan, respectively. Table 1 reported the subject demographics of patients with clinically suspected MK. The mean age was 48.9 and 51.9 years. Two (3.1%) and 33 patients (14.2%) were agricultural workers. A total of 168 (41 in subtropical, and 127 in tropical Taiwan) corneal scrapes with positive fungal or bacterial identification were consecutively enrolled. Among laboratory confirmed cases of FK or BK, there were significant differences between subtropical and tropical Taiwan in proportion of fungal etiology (14.6% vs. 41.7%, p = 0.002) and bacterial etiology (73.2% vs. 49.6%, p = 0.008). Two hundred twenty-five microbes were laboratory confirmed as fungi or bacteria. In subtropical Taiwan, 45 bacteria and 13 fungi were identified from 41 scrapes; in tropical Taiwan, 98 bacteria and 69 fungi were identified from 127 scrapes. The proportion of fungi was significantly higher in tropical Taiwan (22.4%, vs. 41.3%, p = 0.010, Fig. 1), with FBR of 0.29 in subtropical and 0.70 in tropical Taiwan.

**Table 1:** Demographic characteristics of patients with clinically suspected MK

Characteristics	Subtropical	Tropical	*p*
Patients	64	233	
Age (years, mean ± SD)	48.9 ± 23.1	51.9 ± 20.3	0.307^a^
Gender, M/F	31/33	140/93	0.095
Agricultural workers	2 (3.1%)	33 (14.2%)	*0.015*
Laboratory confirmed cases of fungal or bacterial keratitis	41 (64.1%)	127 (54.5%)	0.172
Bacterial infection	30 (73.2%)	63 (49.6%)	*0.008*
Fungal infection	6 (14.6%)	53 (41.7%)	*0.002*
Bacterial/fungal coinfection	5 (12.2%)	11 (8.7%)	0.543
Identified microorganisms	58	167	
Fungi	13 (22.4%)	69 (41.3%)	*0.010*
Bacteria	45 (77.6%)	98 (58.7%)	*0.010*

Pearson’s chi-square test or Fisher’s exact test for proportions, as appropriate

Italic values represent significant *p* values

^a^Student *t* test for continuous variable in normal distribution

The ocular, systemic, and environmental conditions at the time of corneal scraping were defined as the predisposing factors of each identified microorganism. Among the total 225 identified microorganisms, the most common ocular risk factor was ocular trauma (25.8%), followed by soft contact lens (19.1%), and vegetable or plant exposure (11.6%). The OR between the species of isolates (fungi or bacteria) and the predisposing factors of each microorganism were analyzed using univariate and multivariate logistic regression. Table 2 showed that being identified in tropical area (OR 2.395, 95% *CI* 1.153 to 4.978, *p *= 0.019), previous ocular trauma (OR 2.179, 95% *CI* 1.139 to 4.168, *p *= 0.019), and history of plant or vegetable exposure (OR 4.182, 95% *CI* 1.644 to 10.636, *p *= 0.003) were independent predisposing factors for identification of fungi from bacteria. The collinearity diagnostics confirmed that there was no problem with multi-collinearity.

**Table 2:** Univariate and multivariate logistic regression analysis for identification of fungi from bacteria

Predisposing factors	Fungi	Bacteria	Univariate analysis OR (95% *CI*)	*p*	Multivariate analysis OR (95% *CI*)	*p*
Ocular risk factors						
Soft contact lens	9	34	0.395 (0.179, 0.873)	*0.022*	–	0.177
Ortho-K wearing	0	11	–	0.999		
Trauma	32	26	2.880 (1.558, 5.324)	*0.001*	2.179 (1.139, 4.168)	*0.019*
Cataract surgery	8	13	1.081 (0.428, 2.729)	0.869		
PK surgery	2	7	0.486 (0.099, 2.395)	0.375		
Topical steroids	5	13	0.649 (0.223, 1.892)	0.429		
Anti-glaucoma agents	4	13	0.513 (0.162, 1.628)	0.257		
Ocular surface disease	6	19	1.941 (0.742, 5.075)	0.176	–	0.913
Soil/sand	4	4	1.782 (0.434, 7.324)	0.423		
Vegetable/plant	18	8	4.746 (1.960, 11.492)	*0.001*	4.182 (1.644, 10.636)	*0.003*
Iron rust	4	4	1.782 (0.434, 7.324)	0.423		
Systemic risk factors						
Diabetes mellitus	14	18	1.430 (0.670, 3.052)	0.355		
Autoimmune diseases	1	1	1.753 (0.108, 28.406)	0.693		
End-stage renal disease	1	3	1.736 (0.178, 16.964)	0.635		
Environmental risk factors						
Tropical area	69	98	2.437 (1.223, 4.858)	*0.011*	2.395 (1.153, 4.978)	*0.019*
Dry weather^a^	50	69	1.676 (0.965, 2.910)	0.067	–	0.853
Hot weather^b^	26	47	0.948 (0.530, 1.696)	0.858		
Season						
Spring	21	29	–	0.755		
Summer	19	33	0.795 (0.359, 1.763)	0.572		
Autumn	17	29	0.810 (0.356, 1.840)	0.614		
Winter	25	52	0.664 (0.318, 1.387)	0.276		

*CI*, confidence interval; Ortho-K, orthokeratology; OR, odds ratio; PK, penetrating keratoplasty

Italic values represent significant *p* values

Univariate and multivariate logistic regression. Parameters with a *p* value < 0.2 were included into multivariate regression model

^a^Dry weather indicates the average monthly rainfall was less than 80 mm

^b^Hot weather indicates the average temperature on the admission date was more than 27 °C

The fungal and bacterial isolation were listed in Table 3. In subtropical Taiwan, the most common fungal and bacterial isolates were *Fusarium solani* (25%), *Trichosporon faecale* (25%), *Pseudomonas aeruginosa* (23.1%) and *Corynebacterium* sp. (17.9%). In tropical Taiwan, *Fusarium* spp. (50%) was the predisposing fungus; *Pseudomonas aeruginosa* (29.3%) and *Propionibacterium acnes* (13.3%) were the predisposing bacteria.

**Table 3:** Mycology and bacteriology of the patients with definite isolation from corneal scraping

Microorganism	Subtropical	Tropical
	n (%)	n (%)
Total fungal isolates	8	24
Filamentary fungal isolates		
*Acremonium (Cephalosporium)* spp.	0 (0.0)	2 (8.3)
*Aspergillus* sp.	0 (0.0)	1 (4.2)
*Aspergillus versicolor*	1 (12.5)	0 (0.0)
*Beauveria*	0 (0.0)	1 (4.2)
*Bipolaris*	0 (0.0)	1 (4.2)
*Curvularia* spp.	1 (12.5)	3 (12.5)
*Drechslera* sp	1 (12.5)	0 (0.0)
*Fonsecaea* sp.	0 (0.0)	1 (4.2)
*Fusarium solani*	2 (25.0)	5 (20.8)
*Fusarium* spp.	0 (0.0)	7 (29.2)
*Penicillium* sp.	1 (12.5)	0 (0.0)
*Phialophora* spp.	0 (0.0)	1 (4.2)
*Sepedonium* sp.	0 (0.0)	1 (4.2)
*Trichosporon faecale*	2 (25.0)	0 (0.0)
Subtotal	8 (100.0)	23 (95.8)
Yeasts		
*Candida parapsilosis*	0 (0.0)	1 (4.2)
Subtotal	0 (0.0)	1 (4.2)
Total bacterial isolates	39	75
Gram positive organism		
*Actinomyces* sp.	0 (0.0)	1 (1.3)
*Aerococcus* sp.	1 (2.6)	0 (0.0)
*Bacillus* spp.	1 (2.6)	2 (2.7)
*Corynebacterium* sp	7 (17.9)	2 (2.7)
*Kocuria kristinae*	0 (0.0)	1 (1.3)
*Mycobacterium abscessus*	0 (0.0)	1 (1.3)
*Mycobacterium gordonae*	1 (2.6)	0 (0.0)
*Mycobacterium szulgai*	0 (0.0)	1 (1.3)
*Propionibacterium acnes*	4 (10.3)	10 (13.3)
*Staphylococci*, CoNS	2 (5.1)	2 (2.7)
*Staphylococcus aureus*, MRSA	0 (0.0)	3 (4.0)
*Staphylococcus aureus*, MSSA	6 (15.4)	4 (5.3)
*Staphylococcus capitis*	0 (0.0)	1 (1.3)
*Staphylococcus cohnii* ssp. *urealyticus*	0 (0.0)	1 (1.3)
*Staphylococcus epidermidis*, MRSE	3 (7.7)	4 (5.3)
*Staphylococcus saprophyticus*	0 (0.0)	1 (1.3)
*Streptococcus* B Group non AABD	0 (0.0)	1 (1.3)
*Streptococcus salivarius*	0 (0.0)	1 (1.3)
*Viridans group streptococci*	1 (2.6)	0 (0.0)
Subtotal	26 (66.7)	36 (48.0)
Gram negative organism		
*Alcaligenes xylosoxidans*	0 (0.0)	1 (1.3)
*Citrobacter koseri*	0 (0.0)	2 (2.7)
*Comamonas testosteroni*	0 (0.0)	0 (0.0)
*Enterobacter cloacae complex*	0 (0.0)	1 (1.3)
*Enterococcus faecalis*	0 (0.0)	1 (1.3)
*Haemophilus influenzae*	0 (0.0)	1 (1.3)
*Klebsiella* spp.	0 (0.0)	1 (1.3)
*Moraxella catarrhalis*	1 (2.6)	0 (0.0)
*Neisseria elongata*	1 (2.6)	0 (0.0)
*Proteus mirabilis*	0 (0.0)	2 (2.7)
*Pseudomonas aeruginosa*	9 (23.1)	22 (29.3)
*Ralstonia pickettii*	1 (2.6)	0 (0.0)
*Serratia marcescens*	1 (2.6)	5 (6.7)
*Stenotrophomonas maltophilia*	0 (0.0)	3 (4.0)
Subtotal	13 (33.3)	39 (52.0)

CoNS, coagulase-negative *Staphylococci*; MRSA, methicillin-resistant *Staphylococcus aureus*; MSSA, methicillin-sensitive *Staphylococcus aureus*; MRSE, methicillin-resistant *Staphylococcus epidermidis*

## Discussion

This prospective multi-center study found a higher fungal identification rate in tropical area as compared to subtropical area. The main predisposing factor was plant exposure. We found that living in tropical area was also an independent risk factor for identification of fungus, which indicated FK was a climate-sensitive disease.

Many previous studies have emphasized the role of geographic, latitudinal, and seasonal variations in MK [4, 7, 18]. However, there was limited comparative study on the relation between specific microorganisms and corresponding climate zone. Our findings suggested that fungal identification was more common in tropical Aw than subtropical Cfa climate area. To explain this climate variation, we focused on meteorological factors (season, temperature, precipitation) and human factors (harvesting, income status). The temperature in tropical and subtropical area was, on average, higher than 18 °C in winter. On the contrary, the monthly rainfall of winter was significantly less in tropical area. Previous review showed the FBR in subtropical area was less than tropical area [8]. Leck et al. found a higher incidence of fungal keratitis during dry and windy seasons than wet and humid seasons [4]. As in these other studies, the longer dry season in tropical Taiwan in winter may have contributed to the increased identification of fungi, with a fungal to bacterial ratio (FBR) of 0.70 and 0.29 in tropical and subtropical Taiwan, respectively.

**Fig. 2:**
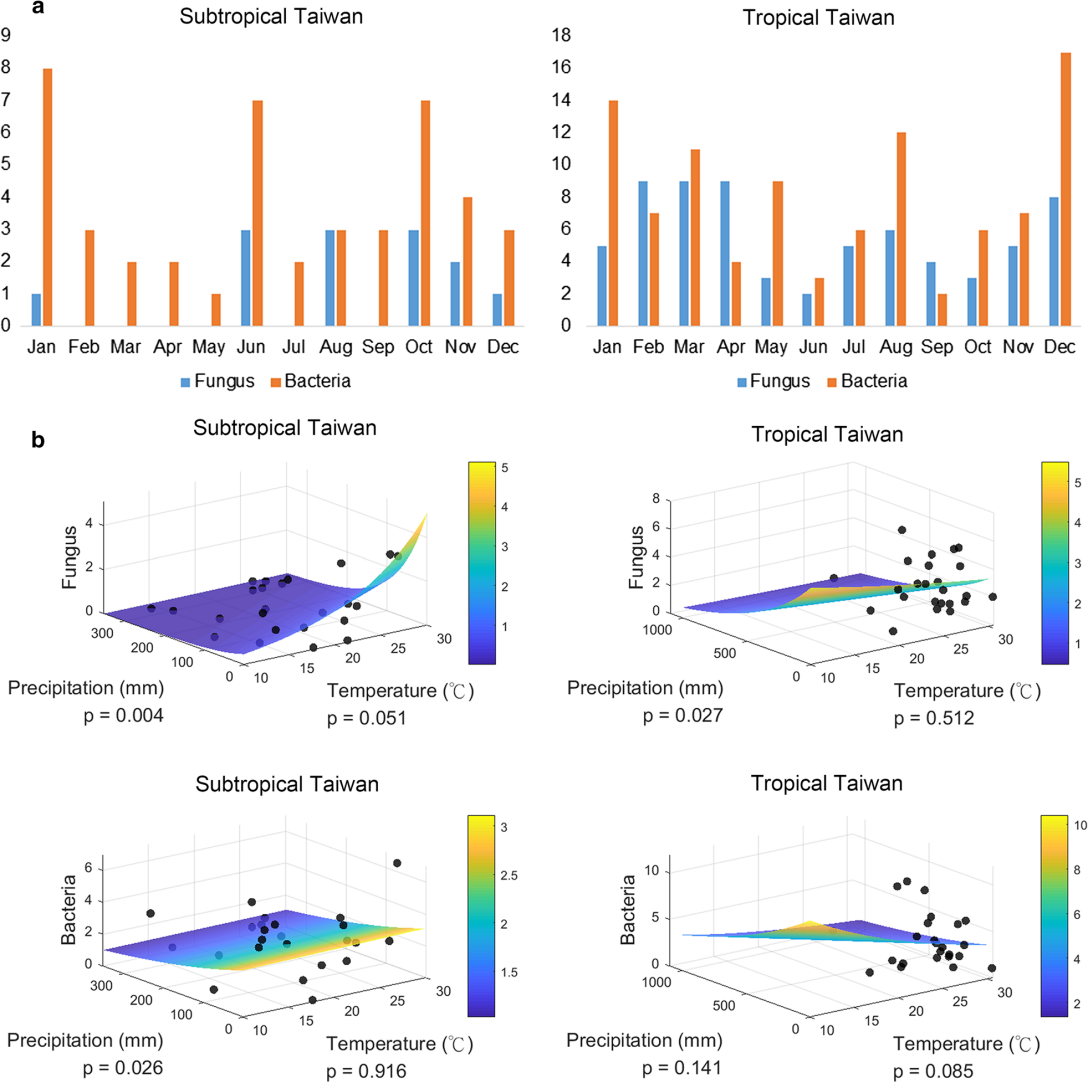
Number of Identified Fungi and Bacteria Distribution. **a** Distribution by month when corneal scraping was performed, in a 2-year period. **b** Three-dimensional scatterplots and Poisson regression surface of number of fungal and bacterial identification, average monthly precipitation and temperature

In our analysis, the possible main determinant meteorological factors for higher fungal isolation were low precipitation and high temperature. The number distribution of fungi and bacteria identified by month in a 2-year period was demonstrated (Fig. 2a). In tropical Taiwan, fungal identification was in a bimodal distribution with a major peak in March and a minor peak in August. There was a statistically significant positive correlation between fungal identification number and dry weather in both subtropical (*p *= 0.004) and tropical area (*p *= 0.027). In subtropical area, fungal identification number was marginally related to hot weather (*p *= 0.051) (Fig. 2b). In contrast, bacterial identification was more frequent in dry weather in subtropical area (*p *= 0.026). Similar seasonal pattern was seen in a recent study conducted in south India (Aw climate type, with dry summer similar to tropical Taiwan) which reported there was a significant 6-month cycle of FK with 2 peaks in July and January; conversely, there was no significant seasonal variation in BK [19].

In terms of low precipitation, previous study showed increasing frequencies of FK during dry and windy seasons [20]. On the contrary, recent study in north India reported more FK noted during post-monsoon season with high humidity [21]. Our study showed that in winter, with the definition of dry months as an average precipitation of less than 80 mm, dry months were more associated with isolation of fungi (OR 9.022, 95% *CI* 1.125 to 72.345, *p *= 0.038). A one-millimeter decrease in monthly precipitation significantly increased the OR of fungal identification by 0.978 (95% *CI* 0.958 to 0.999, *p *= 0.042) per case, according to univariate logistic regression. However, this association was not found in other seasons. As compared to subtropical area, the presence of obvious low precipitation in winter was an important meteorological factor for fungal isolation in tropical area.

Regarding high temperature, earlier study reported a higher proportion of fungal keratitis in decreased latitudes of tropical area [4], which corresponds to increased temperature. A study conducted in south India suggested that FK was more frequent in hot and windy weather [22]. However, our results showed that with the definition of hot months as monthly average temperature higher than 27 °C, there was no significant increase in fungal identification during hot months. Possible explanation was that the minimal differences in latitude and corresponding minimal temperature change were not major meteorological factors for greater identification rate of fungus in tropical area.

The occupation-related corneal ulcer was an important cause of fungal keratitis. Agricultural work-associated vegetable exposure and plant-related ocular trauma were reported as the leading causes of FK [8]. In our study population, there were significantly more agriculture workers in tropical than subtropical Taiwan. Moreover, during the windy harvest season in tropical Taiwan, farmers were more susceptible to FK caused by eye trauma by airborne harvest-related particles as suggested by Chang et al. [23]. Recent study conducted in tropical Taiwan reported that the concentration of fungi on the phylloplane increases with higher temperature and lower wind speed [24]. This could explain the higher rate of fungal identification in tropical Taiwan was strongly related to harvesting.

In addition to harvesting, the extent of urbanization should be taken into consideration [8]. The average income of the three main cities in subtropical Taiwan was 1.24 times that of the tropical Taiwan in recent 3 years. Previous study summarized a significantly higher percentage of fungal isolates and lower bacterial isolates in countries with low income [7]. The higher prevalence of fungal identification may be correlated with relatively low-income class in tropical Taiwan.

In our present study, multiple isolates on culture was significantly greater in subtropical area than tropical area (30% vs. 18%, *p *= 0.006). Notably, in tropical area, one case presented with mixed fungal isolates, *Beauveria* and *Candida parapsilosis*. In Western Australia, Gebauer et al. reported similar results with a greater incidence of mixed culture in increasing tropical latitudes, as a result of the climatic variation [25].

Recent epidemiologic study conducted in Asia reported that the incidence of FK was higher in India and China and was lesser in other Asian countries such as Taiwan [13]. In contrast, our results showed a higher FK identification rate in Taiwan. This higher FK identification rate may have been due to the use of combined molecular identification tests that have a higher sensitivity for fungal keratitis as compared to a single culture test [16].

For disease control, protective eyewear usage should be emphasized and implemented during agriculture works or exposed to plants. In addition, since diagnosis of FK is a challenge, we suggest performing a comprehensive molecular diagnosis to effectively increase the diagnostic rate of FK. Ophthalmologists should be more aware of FK when treating patients in tropical area.

There were some limitations in our study. Firstly, the sample size was smaller in subtropical Taiwan than in tropical Taiwan. Secondly, although we included all the cases of presumed MK and constructed a comprehensive laboratory method including PCR and dot assay to identify the pathogens, it was still possible that the causative pathogen was not identified due to insufficient specimen. Thirdly, the identification of non-disease-causing commensal strains might overestimate the frequency of BK in both tropical and subtropical Taiwan. Fourthly, on the contrary, since all patients were from tertiary healthcare centers and might have received previous antibiotics treatment, which potentially underestimate the frequency of BK. In addition, due to the unavailability of wind speed in each case, we could not evaluate the effects of wind speed on FBR. Finally, since the visual acuity data at the end of the treatment were not collected, our study results failed to identify spatial factors predisposing to a poor visual outcome.

## Conclusions

Our results may provide a few clinically important advices for ophthalmologists in similar climate zones. First, we found a higher FK identification rate in tropical area. Second, in addition to ocular trauma and exposure to plants, living in tropical area was an independent risk factor for identification of FK. Knowledge of these epidemiology and predisposing factors of FK can help early diagnosis and tailor empiric treatment. Third, Taiwan, which locates in both the tropical and subtropical zones and has a homogenous healthcare system, provides a good model to investigate the disease patterns between different climate zones. Taken together, our results suggest that FK is a climate-sensitive infectious disease, and Taiwan can provide a model for further clinical trials on these microbiological patterns.

## Data Availability

The data analyzed during this study are available on request from the corresponding author, Ming-Tse Kuo. The data are not publicly available due to it containing information that could compromise the privacy of research participants.

## Abbreviations

AW: Dry winter
Cfa: Warm temperate climate with fully humid
CI: Confidence interval
BK: Bacterial keratitis
DHA: Dot hybridization assay
FBR: Fungal to bacteria rate
FK: Fungal keratitis
IRB: Institutional research ethics board
KOH: Potassium hydroxide
MK: Microbial keratitis
OR: Odds ratio
Ortho-K: Orthokeratology
PCR: Polymerase chain reaction
PK: Penetrating keratoplasty
VIF: Variance inflation factor
